# O.1.1-11 Are stress symptoms associated to health-enhancing physical activity levels in university students? The case of a Belgian French-speaking university in 2022

**DOI:** 10.1093/eurpub/ckad133.087

**Published:** 2023-09-11

**Authors:** Emma Holmberg, Caroline Mertens, Juliette Paume, Katia Castetbon

**Affiliations:** Service d’Information, Promotion, Éducation Santé (SIPES), Research Centre in “Epidemiology, Biostatistics and Clinical Research”, School of Public Health, Université libre de Bruxelles (ULB), Brussels, Belgium; Service d’Information, Promotion, Éducation Santé (SIPES), Research Centre in “Epidemiology, Biostatistics and Clinical Research”, School of Public Health, Université libre de Bruxelles (ULB), Brussels, Belgium; Observatoire de la Vie Étudiante (OVE), Université libre de Bruxelles (ULB), Brussels, Belgium; Emma.Holmberg@ulb.be; Service d’Information, Promotion, Éducation Santé (SIPES), Research Centre in “Epidemiology, Biostatistics and Clinical Research”, School of Public Health, Université libre de Bruxelles (ULB), Brussels, Belgium

## Abstract

**Purpose:**

Starting university is often associated with a decrease in physical activity and an increase in stress levels. In students, higher levels of stress have been associated with lower PA participation. During the pandemic, students’ levels of PA seem to have decreased whilst stress symptoms (SS) have increased. The purpose of this study was to investigate the association between health-enhancing physical activity (HEPA) and SS in students attending a French-speaking university in Brussels in spring 2022.

**Methods:**

Data was collected using an online questionnaire in 1,754 voluntary students. HEPA was measured using the International Physical Activity Questionnaire-Short Form (IPAQ-SF) and SS were assessed using the Stress single item measure. Binary logistic regressions were used to investigate how much HEPA (yes/no) was associated to SS in three categories: minimally to not at all; very stressed; and highly stressed. Models were adjusted for sociodemographic characteristics. All analyses were weighed to calibrate estimates to the entire university population.

**Results:**

Among the study population (females: 59.3%), over a third of students (35.3%) were classified as HEPA active. This prevalence was the lowest amongst those who were “highly” stressed (28.8%), intermediate in those who were “very” stressed (34.0%) and the highest amongst those who were “minimally or not at all” stressed (39.6%) (p = 0.0005). After adjusting for sociodemographic characteristics, compared to those who were “highly” stressed, those who were “minimally or not at all” stressed were more likely to be HEPA active (aOR: 1.30; 95%CI: 1.00-1.70). There was no difference in the likelihood of being HEPA active for those who were “very” stressed (vs. highly stressed: aOR: 1.16; 95%CI: 0.87-1.56). Among the sociodemographic characteristics, sex and year of study were associated to HEPA. Females were less likely to be HEPA active compared to males (aOR: 0.38; 95%CI: 0.30-0.47), whereas undergraduate students were more likely to undertake HEPA than master’s students (1st year: aOR: 1.30; 95%CI: 1.00-1.69, 2nd-3rd year: aOR: 1.33; 95%CI: 1.03-1.72).

**Conclusions:**

These findings indicate an association between HEPA and SS in students attending a French-speaking university in Belgium in 2022. They will enable to inform health promotion initiatives encouraging HEPA at the university.

